# Crystal structure of hydrazine iron(III) phosphate, the first transition metal phosphate containing hydrazine

**DOI:** 10.1107/S2056989015020010

**Published:** 2015-11-04

**Authors:** Renald David

**Affiliations:** a Laboratoire de Réactivité et Chimie des Solides (LRCS), Université de Picardie Jules Verne, CNRS UMR 7314, 33 rue Saint Leu, 80039 Amiens, France

**Keywords:** crystal structure, hydrazine, iron phosphate, isotypism

## Abstract

The title compound, poly[(μ_2_-hydrazine)(μ_4_-phosphato)iron(III)], [Fe(PO_4_)(N_2_H_4_)]_*n*_, was prepared under hydro­thermal conditions. Its asymmetric unit contains one Fe^III^ atom located on an inversion centre, one P atom located on a twofold rotation axis, and two O, one N and two H atoms located on general positions. The Fe^III^ atom is bound to four O atoms of symmetry-related PO_4_ tetra­hedra and to two N atoms of two symmetry-related hydrazine ligands, resulting in a slightly distorted FeO_4_N_2_ octa­hedron. The crystal structure consists of a three-dimensional hydrazine/iron phoshate framework whereby each PO_4_ tetra­hedron bridges four Fe^III^ atoms and each hydrazine ligand bridges two Fe^III^ atoms. The H atoms of the hydrazine ligands are also involved in moderate N—H⋯O hydrogen bonding with phosphate O atoms. The crystal structure is isotypic with the sulfates [Co(SO_4_)(N_2_H_4_)] and [Mn(SO_4_)(N_2_H_4_)].

## Abstract 2

The structure of the title compound can be described as a three-dimensional network of FePO_4_N_2_ octa­hedra resulting from corner-sharing with four PO_4_ tetra­hedra and bonding with two *trans*-arranged hydrazine mol­ecules.

## Chemical context   

During the last century, transition metal phosphates have been studied intensively not only for their rich crystal- and magneto-chemistry (Kabbour *et al.*, 2012 ▸), but also for their various potential applications. For example, NH_4_ *M* ^II^PO_4_·H_2_O phases, where *M* is a transition metal, are used as pigments for protective paint finishes on metals, as fire retardants in paints and plastics but may also be applied as catalysts, fertilizers and magnetic devices (Erskine *et al.*, 1944 ▸; Bridger *et al.*, 1962 ▸; Barros *et al.*, 2006 ▸; Ramajo *et al.*, 2009 ▸). More recently, it was demonstrated by Goodenough and co-workers that in electrodes the presence of PO_4_ groups results in higher positive potentials (Padhi *et al.*, 1997 ▸), leading to an intensive research on LiFePO_4_, one of the most promising materials for the new generation of Li batteries (Ouvrard *et al.*, 2013 ▸).

## Structural commentary   

**Figure 1:**
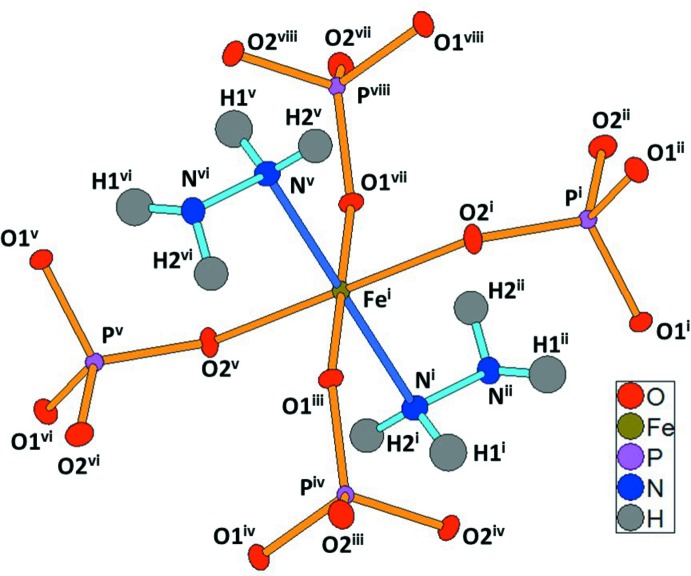
The coordination environment of the Fe^III^ atom in the structure of [Fe(PO_4_)(N_2_H_4_)]. Displacement ellipsoids are drawn at the 50% probability level. [Symmetry codes: (i) *x*, *y*, *z*; (ii) 0.5-*x*, 0.5-*y*; (iii) −*x*, *y* + 0.5, 0.5-*z*; (iv) *x* + 0.5, −*y*, 0.5-*z*; (v) −*x*, −*y*, −*z*; (vi) *x* + 0.5, *y* + 0.5, −*z*; (vii) *x*, 0.5-*y*, *z* + 0.5; (viii) 0.5-*x*, *y*, *z* + 0.5.]

**Figure 2:**
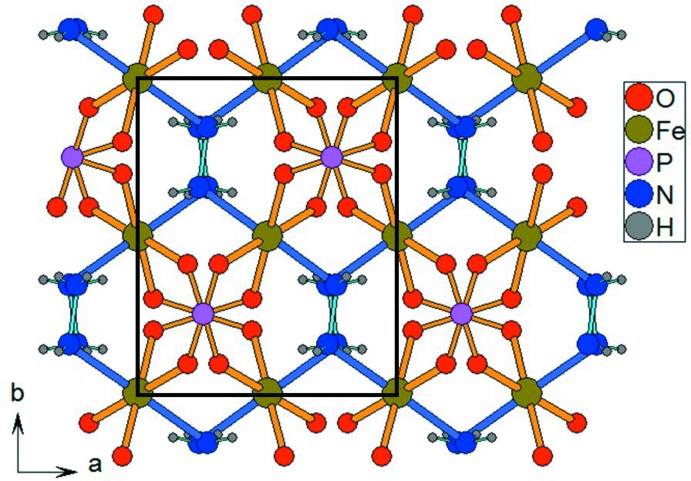
The crystal structure of [Fe(PO_4_)(N_2_H_4_)] in a projection along [001].

The structure of the title compound, [Fe(PO_4_)(N_2_H_4_)], is isotypic with the sulfates [Co(SO_4_)(N_2_H_4_)] and [Mn(SO_4_)(N_2_H_4_)] (Jia *et al.*, 2011 ▸). The Fe^III^ atom is bound to four PO_4_ tetra­hedra and to two N atoms of hydrazine ligands, resulting in a slightly distorted FeO_4_N_2_ octa­hedron (Fig. 1 ▸). The crystal structure consists of a three-dimensional network made up of Fe^III^ atoms which are inter­connected through neutral hydrazine (N_2_H_4_) ligands and phosphate (PO_4_^3−^) anions (Fig. 2 ▸). If the phosphate and sulfate structures are isotypic, the presence of phosphate implies an oxidation state of +III for the transition metal compared to +II for the sulfate analogues. The replacement of sulfate for phosphate leads to a change in the coordination sphere of the metal. These differences are mainly associated with the metal–oxygen bond lengths. The average Fe^III^—O bond length is 1.97 Å for [Fe(PO_4_)(N_2_H_4_)] and the average Co^II^—O bond length is 2.12 Å for [Co(SO_4_)(N_2_H_4_)], whereas the average *M*—N bond lengths involving the N atom of the hydrazine ligand are similar, with values of 2.17 and 2.12 Å, respectively. As a consequence, the FeN_2_O_4_ octa­hedron is more distorted, appearing like an FeO_4_ square additionally bound by two *trans* hydrazine ligands in axial positions.

It should be noted that it seems rather surprising to stabilize Fe^III^ with hydrazine, since the latter is a powerful reducing agent. Efforts are currently underway to obtain the title compound as a pure phase to perform magnetic measurements. It could be a way, by comparison with the results reported for [Co(SO_4_)(N_2_H_4_)] (Jia *et al.*, 2011 ▸), to study the ability of hydrazine to transmit magnetic coupling.

## Supra­molecular features   

**Figure 3:**
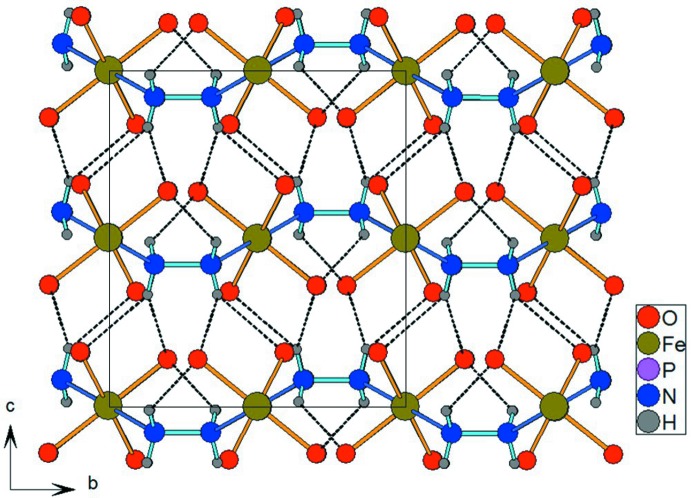
The crystal structure of [Fe(PO_4_)(N_2_H_4_)] in a projection along [100], emphasizing the hydrogen bonding between the components (black dotted lines). P atoms have been omitted for clarity.

The three-dimensional framework structure of [Fe(PO_4_)(N_2_H_4_)] is consolidated by N—H⋯O inter­actions between the hydrazine ligands and phosphate O atoms (Fig. 3 ▸). One of the two hydrogen bonds is bifurcated. Considering the N⋯O distances and the values of the N—H⋯angles (Table 1 ▸), this type of hydrogen bonding can be considered as moderately strong.

## Synthesis and crystallization   

Iron(II) chloride tetra­hydrate (>99.0%, Sigma–Aldrich), hydrazine monohydrate (99+%) and KH_2_PO_4_ (both VWR Inter­national) were used as received without further purification. Iron(II) chloride tetra­hydrate (2 g) was dissolved in water (20 ml) before adding hydrazine monohydrate (2 ml). The obtained solution was stirred for 5 min. Then, KH_2_PO_4_ (11.5 g) was added. After 10 min of stirring for homogenization, the obtained solution (15 ml) was incorporated in a 23 ml autoclave. The autoclave was then heated at 433 K for 10 h before being cooled to room temperature at a rate of 10 K h^−1^. The obtained mixture, consiting of orange crystals of the title phase and yellow crystals of an additional phase, was washed with water. The obtained crystals were very small (around 20 µm) and well isolated from the others. Details of the composition and structure of the yellow crystals will be described in a forthcoming article.

## Refinement details   

Crystal data, data collection and structure refinements are summarized in Table 2 ▸. All H atoms were located in a difference Fourier map and were refined freely with isotropic displacement parameters.

## Supplementary Material

Crystal structure: contains datablock(s) global, I. DOI: 10.1107/S2056989015020010/wm5227sup1.cif

Structure factors: contains datablock(s) I. DOI: 10.1107/S2056989015020010/wm5227Isup2.hkl

CCDC reference: 1432700

Additional supporting information:  crystallographic information; 3D view; checkCIF report

## Figures and Tables

**Table 1:** Hydrogen-bond geometry (Å, °)

*D*—H⋯*A*	*D*—H	H⋯*A*	*D*⋯*A*	*D*—H⋯*A*
N—H1⋯O1^i^	0.85 (3)	2.36 (2)	3.086 (2)	144 (2)
N—H1⋯O2^ii^	0.85 (3)	2.27 (3)	2.974 (2)	141 (2)
N—H2⋯O1^iii^	0.85 (3)	2.19 (3)	2.873 (2)	137 (2)

Symmetry codes: (i) 
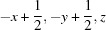
; (ii) 
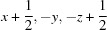
; (iii) 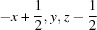.

**Table 2:** Experimental details

Crystal data	
Chemical formula	[Fe(PO_4_)(N_2_H_4_)]
*M*_r_	182.87
Crystal system, space group	Orthorhombic, *P* *c* *c* *n*
Temperature (K)	293
*a*, *b*, *c* (Å)	6.3114 (13), 7.6680 (15), 8.6485 (18)
*V* (Å^3^)	418.55 (15)
*Z*	4
Radiation type	Mo *K*α
μ (mm^−1^)	3.89
Crystal size (mm)	0.05 × 0.03 × 0.03

Data collection	
Diffractometer	Bruker APEXII CCD
Absorption correction	Multi-scan (*SADABS*; Bruker, 2013 ▸)
*T*_min_, *T*_max_	0.668, 0.746
No. of measured, independent and observed [*I* > 3σ(*I*)] reflections	13820, 601, 457
*R*_int_	0.065
(sin θ/λ)_max_ (Å^−1^)	0.717

Refinement	
*R*[*F* ^2^ > 3σ(*F* ^2^)], *wR*(*F* ^2^), *S*	0.020, 0.027, 1.46
No. of reflections	601
No. of parameters	47
H-atom treatment	All H-atom parameters refined
Δρ_max_, Δρ_min_ (e Å^−3^)	0.40, −0.33

Computer programs: *APEX2*and *SAINT* (Bruker, 2013 ▸), *SUPERFLIP* (Palatinus & Chapuis, 2007 ▸), *JANA2006* (Petříček *et al.*, 2014 ▸) and *DIAMOND* (Brandenburg & Putz, 2010 ▸).

## supplementary crystallographic information

### Crystal data

**Table:**

[Fe(PO_4_)(N_2_H_4_)]	*F*(000) = 364
*M**_r_* = 182.87	*D*_x_ = 2.902 Mg m^−^^3^
Orthorhombic, *P**c**c**n*	Mo *K*α radiation, λ = 0.71073 Å
Hall symbol: -P 2ab 2ac	Cell parameters from 2128 reflections
*a* = 6.3114 (13) Å	θ = 4.2–26.9°
*b* = 7.6680 (15) Å	µ = 3.89 mm^−^^1^
*c* = 8.6485 (18) Å	*T* = 293 K
*V* = 418.55 (15) Å^3^	Parallelepiped, orange
*Z* = 4	0.05 × 0.03 × 0.03 mm

### Data collection

**Table:**

Bruker APEXII CCD diffractometer	457 reflections with *I* > 3σ(*I*)
Radiation source: X-ray tube	*R*_int_ = 0.065
phi scan	θ_max_ = 30.6°, θ_min_ = 4.2°
Absorption correction: multi-scan (*SADABS*; Bruker, 2013)	*h* = −9→8
*T*_min_ = 0.668, *T*_max_ = 0.746	*k* = −10→10
13820 measured reflections	*l* = −12→12
601 independent reflections	

### Refinement

**Table:**

Refinement on *F*	0 constraints
*R*[*F*^2^ > 2σ(*F*^2^)] = 0.020	All H-atom parameters refined
*wR*(*F*^2^) = 0.027	Weighting scheme based on measured s.u.'s *w* = 1/(σ^2^(*F*) + 0.0001*F*^2^)
*S* = 1.46	(Δ/σ)_max_ = 0.006
601 reflections	Δρ_max_ = 0.40 e Å^−^^3^
47 parameters	Δρ_min_ = −0.33 e Å^−^^3^
0 restraints	

### Fractional atomic coordinates and isotropic or equivalent isotropic displacement parameters (Å^2^)

**Table:**

	*x*	*y*	*z*	*U*_iso_*/*U*_eq_	
O1	−0.0604 (2)	0.30096 (17)	0.36016 (16)	0.0093 (4)	
Fe	0	0	0	0.00652 (11)	
P	−0.25	0.25	0.25868 (8)	0.00567 (17)	
O2	−0.1898 (2)	0.09269 (19)	0.15978 (16)	0.0111 (4)	
N	0.2656 (3)	0.1556 (2)	0.0781 (2)	0.0103 (5)	
H1	0.306 (4)	0.132 (3)	0.169 (3)	0.024 (7)*	
H2	0.364 (4)	0.139 (3)	0.012 (3)	0.020 (7)*	

### Atomic displacement parameters (Å^2^)

**Table:**

	*U*^11^	*U*^22^	*U*^33^	*U*^12^	*U*^13^	*U*^23^
O1	0.0089 (6)	0.0090 (7)	0.0099 (7)	0.0001 (5)	−0.0032 (5)	−0.0013 (5)
Fe	0.00692 (19)	0.0059 (2)	0.00678 (19)	−0.00013 (13)	0.00010 (16)	−0.00054 (16)
P	0.0059 (3)	0.0053 (3)	0.0057 (3)	−0.0001 (3)	0	0
O2	0.0135 (6)	0.0096 (7)	0.0101 (7)	0.0003 (5)	0.0030 (6)	−0.0033 (6)
N	0.0110 (8)	0.0074 (8)	0.0123 (9)	−0.0017 (7)	−0.0012 (8)	0.0010 (7)

### Geometric parameters (Å, º)

**Table:**

O1—Fe^i^	1.9843 (14)	P—O2^iii^	1.5268 (15)
O1—P	1.5346 (15)	N—N^iv^	1.461 (2)
Fe—O2	1.9621 (15)	N—H1	0.85 (3)
Fe—O2^ii^	1.9621 (15)	N—H2	0.85 (3)
P—O2	1.5268 (15)		
			
Fe^i^—O1—P	133.97 (8)	O1—P—O2^iii^	108.25 (7)
O1^v^—Fe—O1^vi^	180.0 (5)	O1^iii^—P—O2	108.25 (7)
O1^v^—Fe—O2	88.09 (6)	O1^iii^—P—O2^iii^	109.13 (7)
O1^v^—Fe—O2^ii^	91.91 (6)	O2—P—O2^iii^	111.85 (9)
O1^vi^—Fe—O2	91.91 (6)	Fe—O2—P	146.37 (9)
O1^vi^—Fe—O2^ii^	88.09 (6)	N^iv^—N—H1	104.6 (18)
O2—Fe—O2^ii^	180.0 (5)	N^iv^—N—H2	104.2 (18)
O1—P—O1^iii^	110.23 (8)	H1—N—H2	112 (2)
O1—P—O2	109.13 (7)		

Symmetry codes: (i) −*x*, *y*+1/2, −*z*+1/2; (ii) −*x*, −*y*, −*z*; (iii) −*x*−1/2, −*y*+1/2, *z*; (iv) −*x*+1/2, −*y*+1/2, *z*; (v) −*x*, *y*−1/2, −*z*+1/2; (vi) *x*, −*y*+1/2, *z*−1/2.

### Hydrogen-bond geometry (Å, º)

**Table:**

*D*—H···*A*	*D*—H	H···*A*	*D*···*A*	*D*—H···*A*
N—H1···O1^iv^	0.85 (3)	2.36 (2)	3.086 (2)	144 (2)
N—H1···O2^vii^	0.85 (3)	2.27 (3)	2.974 (2)	141 (2)
N—H2···O1^viii^	0.85 (3)	2.19 (3)	2.873 (2)	137 (2)

Symmetry codes: (iv) −*x*+1/2, −*y*+1/2, *z*; (vii) *x*+1/2, −*y*, −*z*+1/2; (viii) −*x*+1/2, *y*, *z*−1/2.
